# Periploca forrestii saponin ameliorates CIA via suppressing proinflammatory cytokines and nuclear factor kappa-B pathways

**DOI:** 10.1371/journal.pone.0176672

**Published:** 2017-05-02

**Authors:** Chunmei Bao, Yingqin Liu, Xin Sun, Congcong Xing, Luting Zeng, Guangchen Sun

**Affiliations:** 1 Department of Nephrology, Guilin Medical University, Guilin, Guangxi, China; 2 Biotechnology College, Guilin Medical University, Guilin, Guangxi, China; 3 Medical College, Xiamen University, Xiamen, Fujian, China; 4 Pharmacy College, Guilin Medical University, Guilin, Guangxi, China; China Medical University, TAIWAN

## Abstract

**Objective:**

Periploca forrestii Schltr has been used as a Chinese folk medicine for the treatment of rheumatism, arthralgia and fractures. However, the anti-arthritic activity of Periploca forrestii saponin (PFS) and the active compound has still not been revealed. This study aimed to investigate the protective effects and mechanisms of PFS on collagen type II (CII) collagen-induced arthritis (CIA) mice. We sought to investigate whether PFS and Periplocin could regulate osteoclastogenesis, and if so, further investigation on its mechanism of action.

**Methods:**

Arthritis was induced in female BALB/c mice by CIA method. PFS was administered at a dose of 50 mg/kg body weight once daily for five weeks. The effects of treatment in mice were assessed by histological and biochemical evaluation in sera and paws. Anti-osteoclastogenic action of PFS and Periplocin was identified using an osteoclast formation model induced by RANKL.

**Results:**

PFS ameliorated paw erythema and swelling, inhibited bone erosion in ankle joint histopathological examination. PFS treatment resulted in decreased IgG2a, and increased IgG1 levels in the serum of CIA mice. Decreased TNF-α, and increased interleukin (IL)-4 and IL-22 levels were also found in PFS-treated mice. PFS inhibited the I-κBα phosphorylation, blocked nuclear factor (NF)-κB/p65 phosphorylation and abrogated AP-1/c-Fos activity. PFS downregulated toll-like receptor (TLR) 4, STAT3 and MMP-9 expression in CIA mice and RANKL-induced osteoclastogenesis. PFS and Periplocin inhibited RANKL-induced osteoclast formation in a dose dependent manner within nongrowth inhibitory concentration, and PFS decreased osteoclastogenesis-related marker expression, including cathepsin K and MMP-9.

**Conclusion:**

This study revealed that the protective mechanism of PFS on CIA was associated with regulatory effects on proinflammatory factors and further on the crosstalk between NF-κB and c-Fos/AP-1 in vivo and in vitro. Therefore, PFS is a promising therapeutic alternative for the treatment of RA, evidencing the need to conduct further studies that can identify their active components in treating and preventing RA.

## Introduction

Rheumatoid arthritis (RA) is a chronic autoimmune disease with 1% prevelance in industrialized countries. It comprises a syndrome of joint pain, swelling, stiffness and symmetrical synovitis of diarthrodial joint that lead to functional decline and deformity. RA pathology is relevant to the immune system and skeletal system, its etiology and pathogenesis remain not entirely clear, and many cell types, such as fibroblasts, T cells, B cells, and osteoclasts (OCs), have been implicated. These inflammatory cells infiltrate the synovium and are further activated to release many cytokines, autoantibodies, and matrix metalloproteinases (MMPs), leading to cartilage and bone destruction [1]. Pro-inflammatory cytokines such as tumor necrosis factor (TNF)-α, IL-6, IL-1β and interferon (IFN)-γ play significant roles in mediating joint inflammation [2,3]. These cytokines are expressed in the arthritic synovium in RA and induce the expression of receptor activator of nuclear factor kappa B ligand (RANKL). The binding of RANKL to its receptor, RANK, triggers the activation of signal transducer and activator of transcription 3 (STAT3), and transcription factors including nuclear factor-κ B (NF-κB), activator protein-1 (AP-1), and nuclear factor of activated T-cells (NFATc1) [4,5]. The activation of these transcription factors directly stimulates the expression of a number of osteoclastogenesis-specific genes, including Tartrate-resistant acid phosphatase (TRAP), MMP-9, NFATc1 and cathepsin K, leading to osteoclast differentiation and bone resorption [6, 7].

Historically, natural products have yielded a variety of therapeutic agents. Perloca Forrestii Sehltr, the whole vine of an asclepiadaceae plant, has been used in folk medicine for the treatment of a variety of inflammatory disorders including RA. Periploca forrestii mainly contains cardiac glycosides, flavonoids and steroids. Periplocin is one of cardiac glycosides extracted from Periploca forrestii, several studies have reported its effects on the various heart diseases [8–11]. Recent studies also suggest that Periplocin from cortex periplocae can suppress cell growth in colon cancer cells, lung cancer cells and hepatocellular Carcinoma Cells [12–14].

In this study, we have examined the effect of PFS on the production of proinflammatory cytokines and transcription factors in the paw tissue in a mice CIA model. We also observed that PFS and Periplocin decrease osteoclastogenesis with modulating NF-κB pathway protein expression.

## Materials and methods

### Animals

BALB/c mice (6–8 week old) with a mean weight of 25–30 g were obtained from Laboratory Animal Center of Guilin medical school, China. Mice were housed in an appropriate environment with an air-filtering system, and maintained on a 12 h light/dark cycle. Animal experiments were approved by the Committee of Ethics on Animal Experiment at Committee of Guilin Medical University, China. All techniques were performed under isoflurane anesthesia, and all efforts were made to minimize suffering.

### Reagents and antibodies

Periplocin was purchased from State General Administration of the People's Republic of China for Quality Supervision and Inspection and Quarantine. The recombinant human RANKL was purchased from Peprotech Biotechnology. M-CSF was purchased from eBioscience. STAT3, phospho-I-κBα, phospho-p65, c-Fos, cathepsin K, NFATc1, Goat Anti-Mouse IgG1-HRP and Goat Anti-Mouse IgG2a-HRP antibodies were purchased from Santa Cruz Biotechnology. I-κBα, TLR4 and MMP-9 antibodies were purchased from Abcam. Phospho-STAT3 Tyr705 antibodies was obtained from Signalway. Alfa-MEM medium, fetal bovine serum and trypsin were purchased from Gibco Inc. TNF-α, IFN-γ, IL-1β, IL-4 and IL-22 ELISA kit were purchased from ebioscience. DMSO and MTT were purchased from Sigma-Aldrich Shanghai, China.

### Collection of Periploca forrestii and saponin extract process

Periploca forrestii were collected from Guangxi province China. The powder obtained from mincing dried roots was extracted for 2 h with 70% ethanol. The extract was re-extracted twice following same procedure and then filtered. The supernatant was partitioned with petroleum ether and then butanol (1:1). The butanol extract was concentrated and dried to obtain PFS. To study the anti-arthritic activity, PFS was given daily by oral gavage at a dose of 50 mg/kg body weight, which was translated from human studies, for five weeks.

### Induction of arthritis

CIA in mice was induced according to the methodology with minor changes [15–18]. Thirty female BALB/c mice were randomly divided into the following three groups: Normal control group = normal mice; CIA Control group = Chicken type II collagen -induced arthritis mice; FPS group = PFS 50 mg/kg-treated CIA mice. Chicken type II collagen (CII) was purchased from Sigma-Aldrich and was solubilized overnight at 4°C in 0.05 M acetic acid at a concentration of 4 mg/ml. The solution was emulsified in an equal volume of Complete Freund's Adjuvant containing 1 mg/ml (heat-denatured mycobacterium). For the induction of CIA, 6–8 week-old female BALB/c mice were immunized with 50 μg of type II chicken collagen by intradermal injection into the subplantar of both hind paws. On day 16, a booster dose of 50 μg CII emulsified in CFA containing 2 mg/ml heat-denatured mycobacterium was administered. Ankle joint Inflammation was apparent 4–8 days after the first dose, thickness of hindlimb were measured with a 0.01 mm precision dial gauge (Peacock Japan) each 3–5 d interval. Arthritis severity was determined using a standard visual scoring system based on the degree of edema and erythema ranging from 0 to 4 for each paw [19]. To explore the effects of PFS on CIA, BALB/c mice were fed PFS daily, commencing on day -7 before primary immunization and keep feeding for 5 weeks. Vehicle mice received water alone.

### Histology and immunohistochemistry

On Day 28, the right hind limbs of BALB/c mice were harvested and dissected, immersed in 4% buffered formalin for fixation for at least 24 h. Next, the tissues were decalcified in 10% EDTA for up to 1 months, then embedded in paraffin. Tissue sections (5 μm) were prepared by mounted on adhesive slides, and then stained with HE, safranin O, toluidine blue O and TRAP staining. Slides for TRAP staining were incubated at 37°C for 30 min in napthol AS-MX phosphate (0.1 mg/ml) in acetate-tartrate buffer (200 mM sodium acetate, 100 mM potassium sodium tartrate, pH 5.2) and counterstained with 0.02% Fast Green for 30 sec. Synovial inflammation and cartilage erosion were defined on HE staining were quantified on each slide using a three-point scale ranging from 0 to 3 as previously described [19]. Other sections were scored for loss of safranin O staining as a measure of cartilage proteoglycan depletion using a scale from 0 to 3 as described elsewhere [20]. For immunohistochemistry, the activity of endogenous peroxidase was blocked with 3% hydrogen peroxide (H_2_O_2_) in methanol. Tissues were first incubated with primary antibodies against Cathepsin K, MMP-9, STAT3, and p-p65, overnight at 4°C. A secondary peroxidase antibody was added and incubation continued for a further 1 h. Final colored products were developed using the chromogen diaminobenzidine and the sections examined under a photomicroscope (Olympus, Tokyo, Japan). Positive control tissues were used to test the specificity for each primary antibody. Staining for the specific expression marker received an optical density (O.D) determination using ImageJ software. Again, the sample identity was withheld to prevent bias.

### Enzyme-linked immunosorbent assay (ELISA)

After four-week experiment, sera were collected from BALB/c mice and stored at -80°C until analysis. 50 μl of CII (5 μg/ml) in PBS were coated onto each well of an ELISA plate overnight at 4°C. The plates were blocked using 1% BSA at 37°C for 1 h. Sera from immunized mice were loaded into the wells and incubated for 2 h at room temperature. The plates were washed, 50 μl of HRP conjugated antibodies was added to each well for 1 h at room temperature. Serum dilution was optimized. HRP activity was measured using tetramethylbenzidine solution (eBioscience). Results were analyzed by measuring the absorbance at 450 nm.

Hind paws and ankles were removed from the sacrificed mice, frozen in liquid nitrogen and lysed using RIPA buffer with protease inhibitor cocktail (Sigma chemical). The crude extract was then sonicated for 30 s. The homogenate was centrifuged at 20,000 × g for 15 min, and the resulting supernatant was collected. The levels of inflammatory factors in the supernatant were measured using specific ELISA kits according to the manufacturer's instructions.

### Osteoclastogenesis

For the primary osteoclasts, total bone marrow cells were collected from tibia and femur of 8 week old female mice by flushing the marrow space with α-MEM. After removing the red blood cells with an ammonium-chloride-potassium buffer, cells were cultured for 1 day in α-MEM containing 10% fetal bovine serum. Nonadherent cells were collected and further cultured with M-CSF in α-MEM containing 10% FBS. After 3 days, culture medium was removed and adherent bone marrow macrophages (BMMs) were used for osteoclast differentiation. BMMs were further incubated with 50 ng/ml M-CSF, 100 ng/ml RANKL, and various concentrations of PFS and Periplocin, for 6 days. On day 3, the medium was replaced with fresh medium containing M-CSF, RANKL, and PFS and Periplocin.

After 6 days’ culture, the cells were washed twice with PBS and then fixed with 4% paraformaldehyde for 10 min. Cells were stained with TRAP at 37°C for 1 h as prescribed previously, and counterstained with Hematoxylin. Cells were then washed and air dried for photography and counting. TRAP-positive cells with more than three nuclei were defined as osteoclasts.

### Cell viability assay

BBMs prepared as described above were plated in 96-well plate at a density of 1×10^4^ cells/well. The cells were incubated with different concentrations of PFS and Periplocin in triplicate. After 3 days incubation, cells were incubated with 20 μl of MTT (5 mg/ml) for 4 h at 37°C. The medium was removed, the MTT crystals were solubilized with 150 μl DMSO, spectrophotometric absorbance of each sample was then measured at 570 nm.

### Western blot analysis

Paws or cells were homogenized or lysed in RIPA lysis buffer (50 mM Tris-HCl, 150 mM NaCl, 1% Triton X-100 and 1% deoxycholate) supplemented with (15 mM sodium fluoride, 1 mM sodium vanadate, 2 mM sodium pyrophosphate, 1 mM sodium glycerophosphate, 2 mM imidazole) and protease inhibitor mixture (Sigma chemical) and incubated for 20 min at 4°C. The lysate was centrifuged at 12000 r/min for 20 min. The supernatant was stored at -80°C. The levels of target proteins were determined. Cell lysates containing 20 μg of total proteins were separated using 15% SDS-polyacrylamide gel electrophoresis and transferred to polyvinkylidene difluoride membranes. The membranes were blocked in 5% skim milk in TBS buffer (0.1% Tween-20; pH 7.6) at room temperature for 1 h and incubated with the corresponding primary antibodies overnight at 4°C. After washing with TBS, the membranes were incubated with appropriate peroxidase-conjugated secondary antibodies. Immunoreactive proteins were detected using the Molecular Imager ChemiDoc XRS+ Imaging System and Chemiluminescence (Bio-Rad Laboratories Inc., Hercules, CA, USA). The gray values of protein bands were measured using ImageJ.

### Statistical analysis

All experimental results are expressed as mean (mean±SEM) of several independent experiments. Statistical comparisons were made using Student’s t test, or one-way analysis of variance (ANOVA) followed by Student—Neuman—Keuls’s multiple comparison. P values of less than 0.05 were regarded as significant.

## Results

### PFS suppressed CIA

**Fig 1:**
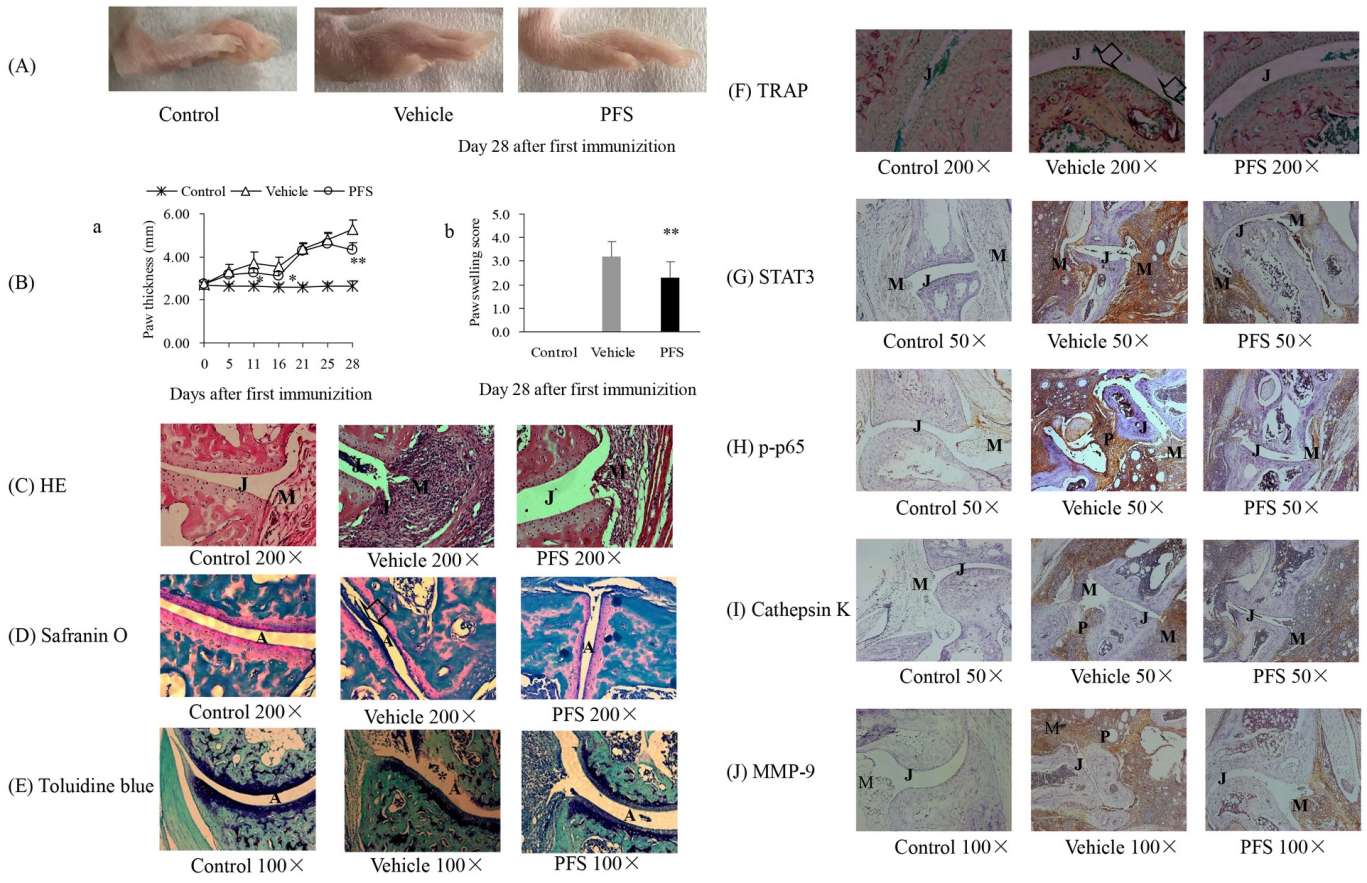
Effect of PFS on developing (prophylactic) collagen-induced arthritis. **(A)** Images of the hindpaw from BALB/c mice. **(B)** a: Change in ankle thickness. b: Joint swelling score on Day 28. The changes in paw thickness were measured every four day. Data are expressed as the mean ± standard error (n = 7–9). Statistical values conducted on days 0–28 for changes in paw thickness compared with vehicle were as follows: *p<0.05, **P < 0.01, compared with the vehicle group, student’s t-test. **(C)** Day 28 paws were harvested, sectioned, and stained with H&E to evaluate for the number of inflammatory cells/erosions. **(D)** Safranin O and fast green staining for cartilage, Extensive proteoglycan depletion (arrowhead). **(E)** Toluidine blue staining to evaluate for cartilage integrity, bone erosions (asterisks). **(F)** TRAP staining for osteoclasts infiltrating the subchondral bone plate up to the cartilage (arrowheads). **(G-J)** Immunohistology for STAT3, p-p65, cathepsin K and MMP-9. A: Articular cartilage; J: Joint space; M: Synovial membrane; P: Pannus formation.

The effect of PFS was evaluated using CIA, a well-established in vivo model of inflammatory joint diseases. Arthritis developed rapidly in mice after the first injection of CFA. By days 25–28 following a booster immunization, significant paw swelling and an increased arthritis index were observed in the model group compared with baseline levels, and these effects plateaued at approximately days 25–28. Mice were administered orally with PFS for 5 weeks from -7 day before the first immunization. Although the arthritis swelling of both vehicle and PFS-treated mice began to increase initially, the swelling of PFS -treated mice were significantly lower than those of vehicle mice on days 11, 21, 28 (Fig 1A and 1B). Arthritis swelling for the group treated with 50 mg/kg of PFS was 4.30 ± 0.36 (mm), against 5.29 ± 0.41(mm) for the vehicle treated group on Day 28. The arthritis score result suggests that PFS inhibits inflammatory reactions in the joints of CIA mice (p < 0.01, Fig 1B).

### PFS improved the histological parameters of CIA mice

Representive image of histology and immunohistochemistry was shown in Fig 1C–1J. HE staining of joint slides indicated that articular capsules in the normal control group appear intact synovial and fibrous membranes and the articular cartilages were thick. By contrast, the synovial membranes of vehicle mice were destroyed and infiltration of inflammatory cells was observed in the synovial tissues, lower hyperplasia and inflammatory cell infiltration in the synovial membrane were observed in PFS-treated mice (Fig 1C and S1 Table). Histological analysis of joints stained with safranin O showed the rough cartilage surfaces and extensive proteoglycan depletion (arrowheads) were noted in vehicle-treated animals, however, the significantly lighter pathological changes were observed in PFS-treated mice compared with mice in the vehicle group (Fig 1D and 1E). Toluidine blue staining showed cartilage erosions (asterisks) were noted along the bone surface in vehicle-treated animals. (Fig 1E and S1 Table). When compared to vehicle mice, TRAP staining osteoclasts failed to resorb the subchondral bone plate up to the noncalcified cartilage at some locations in PFS-treated mice, which suggests that PFS reduces osteoclast infiltration (Fig 1F). Immunohistochemical analysis of the paw sections showed that the reduction of the arthritis-induced MMP-9 and p-p65 expression upon PFS administration occurred in the synovial membrane, and also in the bone pannus (Fig 1G, 1H, 1I and 1J). Whilst the tissue score of STAT3, p-p65, MMP-9 and cathepsin K expressions in both vehicle and PFS-treated mice were higher than those in normal control mice, score levels of STAT3, p-p65, MMP-9 and cathepsin K in PFS-treated mice were significantly less compared with vehicle mice (S2 Table).

### PFS regulated antibody and inflammation mediator levels in CIA mice

**Fig 2:**
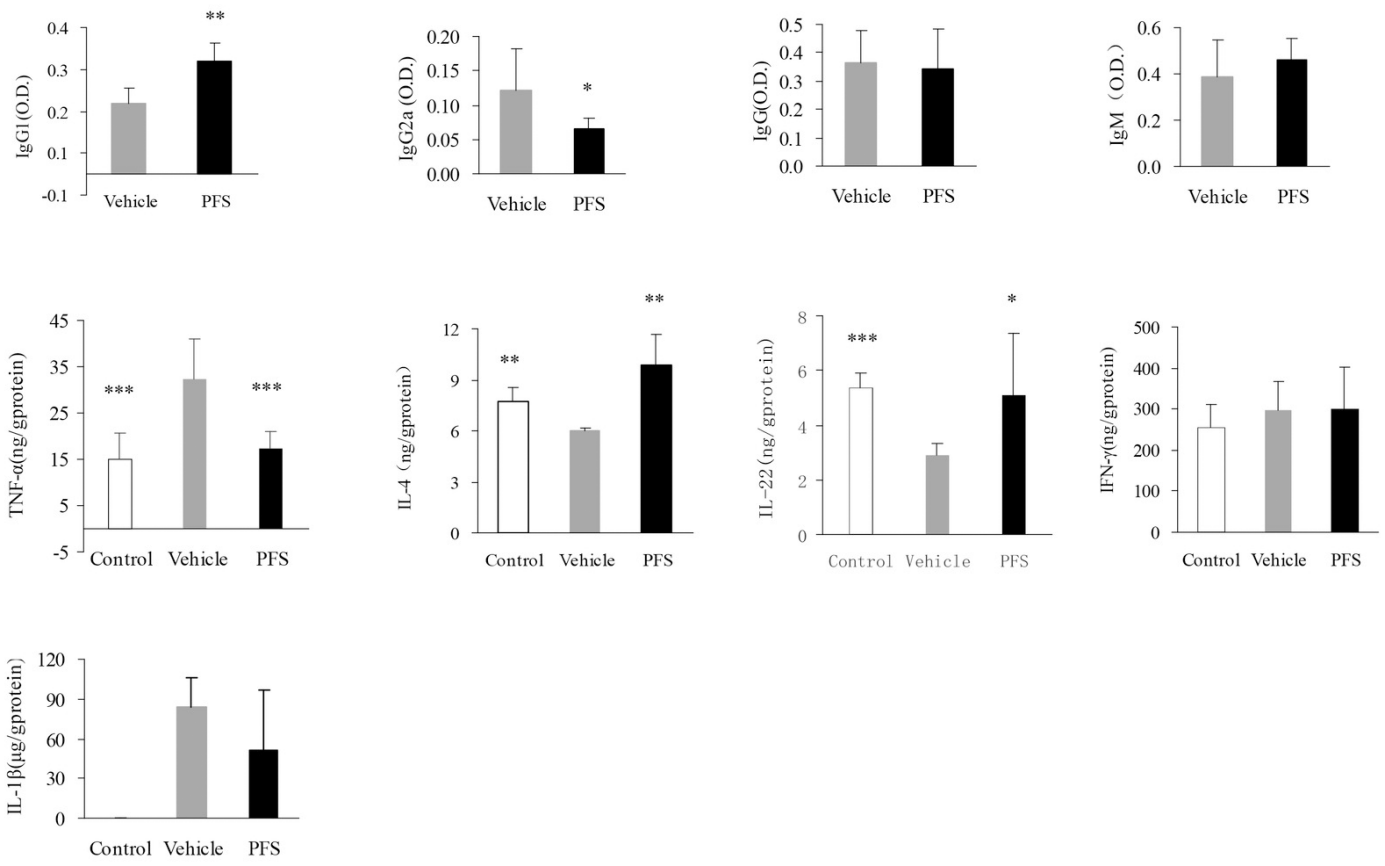
Effect of PFS on serum antibody levels, tissue cytokines levels. Mice CIA was induced as prescribed in Method. Serum samples were collected on day 28 after the start of treatment, chicken CII—specific antibody levels in sera and cytokine levels in lysates of paw were analyzed by ELISA. Data represent 7–9 mice of Mean ± SEM. Statistical analysis was performed using Student’s t-test. *P < 0.05, **P < 0.01 *** P < 0.001versus vehicle group.

To address the potential immunogenicity of PFS, we measured specific antibody responses against CII following 2 times of serial CII injections. Results indicate that CII antigen-specific IgG2a antibodies in PFS-treated mice were dramatically reduced (P < 0.05) when compared to vehicle-fed CIA mice (Fig 2). In contrast, the titers of Th2-dependent CII specific IgG1 antibodies were increased in PFS-treated mice compared with vehicle-treated mice (P < 0.05). These results suggest an important role for PFS in modulating B cell responses, as well as Th1/Th2 balance in the ongoing immune response. No significant difference was observed for IgG or IgM responses in PFS-treated mice compared with vehicle-treated mice.

We then examined the levels of cytokine expression in the inflammatory paws. PFS significantly inhibited TNF-α, but not IL-1β and IFN-γ levels, while increased IL-4 and IL-22 levels (Fig 2).

### PFS suppressed NF-κB signal pathway proteins in CIA mice

In the course of inflammation, immune cells of the innate and/or adaptive immune system are activated, and a variety of different cytokines recruited to the inflammation site are predominantly regulated by NF-κB and other transcription factors including AP-1, NFATs, and STATs.

**Fig 3:**
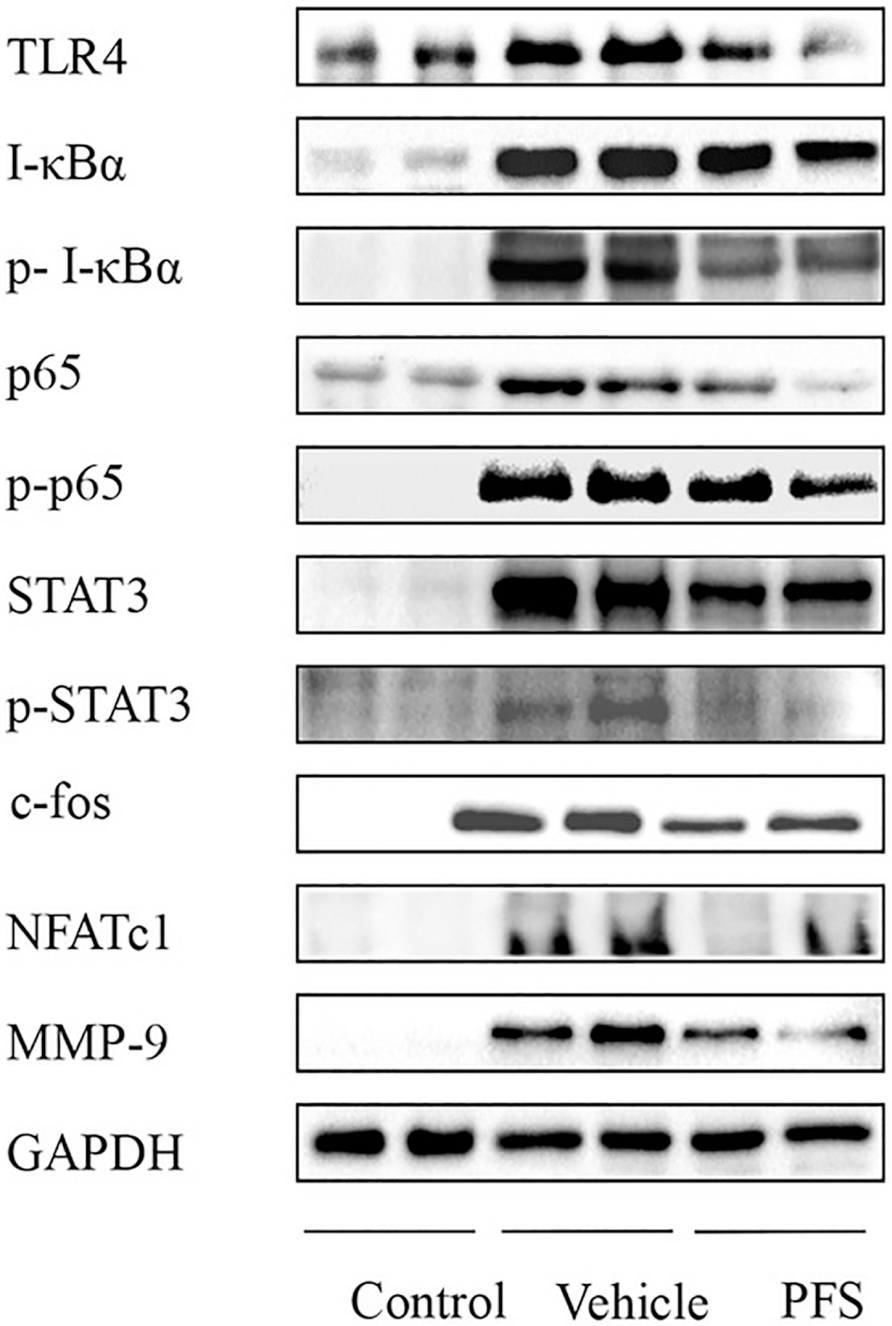
PFS downmodulate the protein expression of NF-κB pathway specifically in the paws. On day 28, mice paws were homogenized, lysates were analyzed by western blotting with antibody against TRL4, I-κBα, p-I-κBα, p65, p-p65, STAT3, p-STAT3, c-Fos, NFATc1 and MMP-9. P: Periplocin.

Western blot demonstrated that PFS inhibited the expressions of TLR4, STAT3, p-STAT3, I-κBα, p-I-κBα, p-p65 and c-Fos, compared to the vehicle group (Fig 3, S1 Fig). However, levels of p50, p65 and NFATc1 in PFS-treated group did not show significantly descent compared with those in the vehicle group. These results indicate that PFS inhibited CIA by suppressing the expression of transcription factors NF-κB, c-Fos, and slightly NFATc1. PFS -treated CIA mice had a significantly lower level of MMP-9 in affected joints than did vehicle-treated mice.

### PFS and Periplocin suppressed RANKL-induced osteoclastogenesis via NF-κB signal pathway

**Fig 4:**
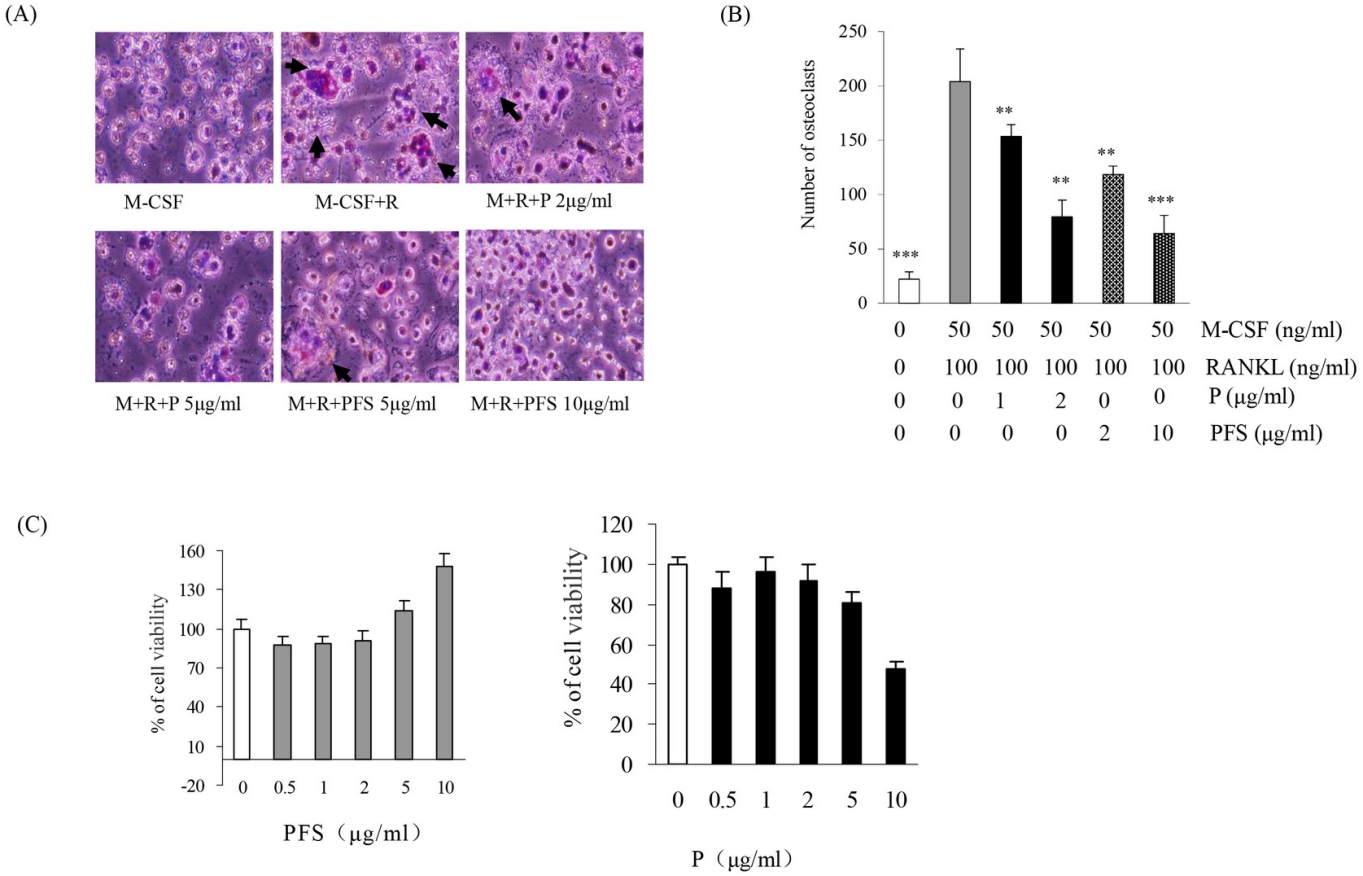
PFS and Periplocin inhibit osteoclast formation in BALB/c mice. (A) BMMs were cultured for 6 days with M-CSF (50 ng/ml) and RANKL (100 ng/ml) in the presence or absence of PFS and Periplocin. Cells were fixed in 3.7% formalin, and stained with TRAP solution. TRAP-positive cells were photographed under a light microscope. TRAP-positive multinucleated osteoclasts (TRAP^+^) with more than three nuclei were counted **(B)**. **(C)** BBMs (1×10^4^ /well) were cultured in 96-well plates for 14 days, incubated with various concentrations of PFS and Periplocin for another 3 days, viability of BMM from bone marrow was measured MTT assay. ** P < 0.01, *** P < 0.001 versus M-CSF + RANKL control, student’s t-test. P: Periplocin.

Since BMMs can differentiate into OCs under RANKL induction, the effect of PFS and Periplocin (S2A Fig) on osteoclast formation was examined. By TRAP staining the number of RANKL-induced osteoclast cells were detected under inverted microscope (Fig 4A). TRAP-positive cells containing three or more nuclei were counted osteoclasts. Notably, OCs treated with PFS and Periplocin exhibited morphological differences with control OCs, being smaller in size and containing fewer numbers of nuclei. Administration of PFS and Periplocin showed a decrease in polynuclear osteoclast formation after RANKL treatments in a concentration-dependent manner (Fig 4B).

Cytotoxic effects of PFS and Periplocin on primary BMMs was examined by the colorimetric MTT assay. As shown in Fig 4C, 0.5, 1, 2, 5 and 10 μg/ml of PFS did not cause any cytotoxic effect on the total cell population. Only Periplocin at 10 μg/ml was found to induce a slight but not significant decrease of viability.

**Fig 5:**
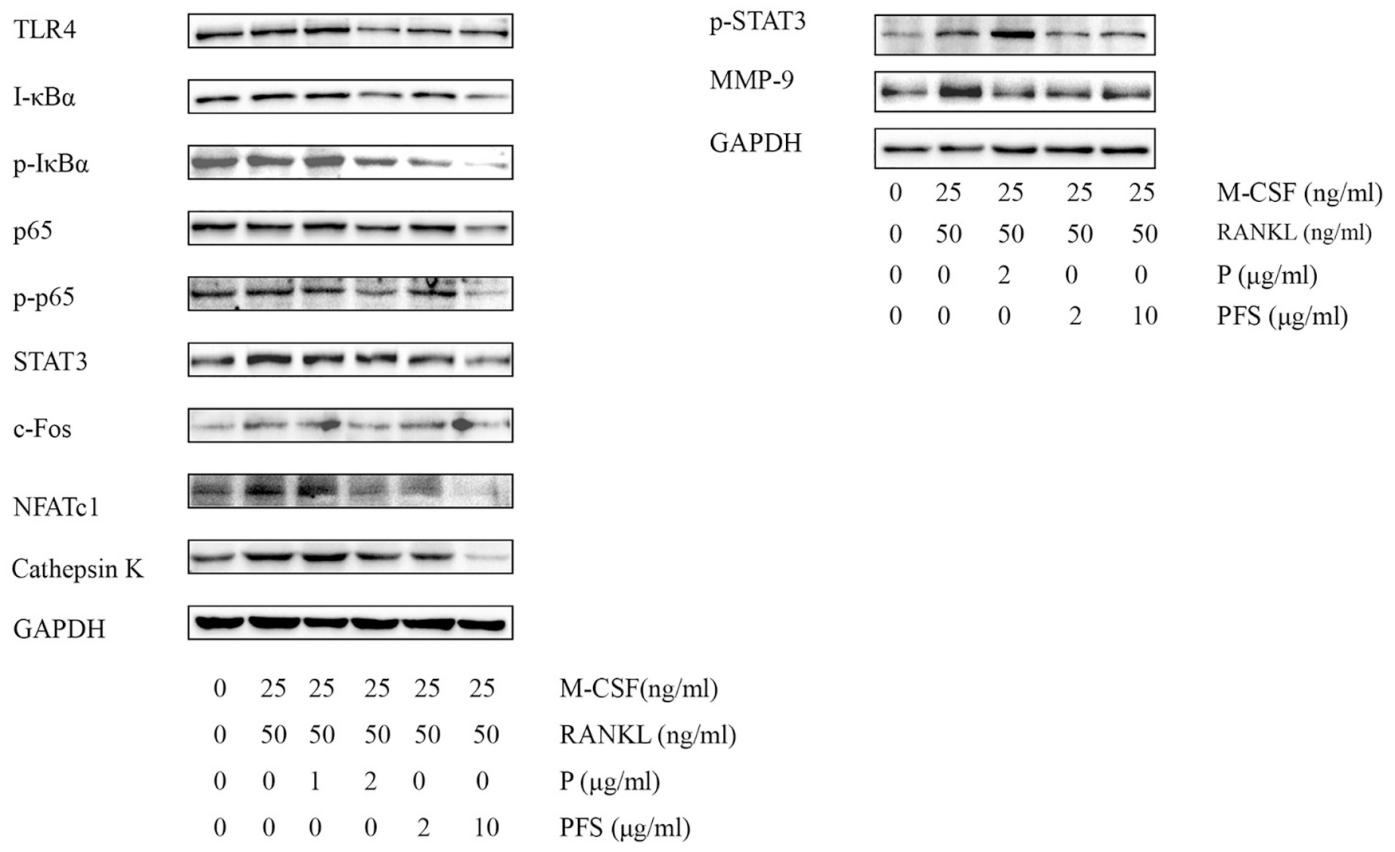
Effect of PFS and Periplocin on RANKL-induced signaling pathway. Bone marrow cells from 6–8 week BALB/c mice were seeded in 10 cm dish plus 25 ng/ml M-CSF. After 3 days incubation, BMMs were then cultured for 3 days in the presence of M-CSF (25 ng/ml) and RANKL (50 ng/ml), then stimulated for 3 days with M-CSF (25 ng/ml) and RANKL (50 ng/ml) in the presence or absence of PFS and Periplocin in the indicated concentrations. Cell lysates were analyzed by western blotting with antibody against TRL4, I-κBα, p-I-κBα, p65, p-p65, STAT3, p-STAT3, c-Fos, NFATc1, MMP-9 and Cathepsin K. P: Periplocin.

In order to explore more insightful the suppression mechanisms of PFS and Periplocin on RANKL-induced osteoclast, we measured the expression of inflammation associated factor (TLR4), transcription factor (STAT3, NF-κB, c-Fos and NFATc1) and proteolytic enzyme (MMP-9 and cathepsin K) expressions in osteoclast formation by Western blotting. Both PFS and Periplocin administration showed a significant reduction of TLR4 and p-p65 activities in a concentration dependent manner, PFS treatment also showed significant inhibition activities on p-I-κBα and p-STAT3 expressions. PFS displayed more strong inhibition activities on c-Fos expression than Periplocin. RANKL induced significant increases in osteoclast marker gene cathepsin K expressions, PFS and Periplocin treatment showed significant decreased activities in a concentration dependent manner (Fig 5, S2B Fig).

## Discussion

Previous studies showed that PFS suppressed Freund’s Complete Adjuvant induced rat arthritis (AIA) by downregulating mRNA levels of IL-6 and TGF-β1 in vivo and reducing joint protein expression levels of phospho-STAT3 and IKKα. Periplocin derived from Periploca forrestii Schltr depressed the mRAN levels of proinflammtory factors in LPS induced AIA splenocytes in vitro [21]. In the current study, we demonstrated that PFS successfully and potently suppresses ongoing experimental arthritis in CIA mice and reduces inflammation-related bone destruction and osteoclastgenesis by inhibiting NF-κB signal pathway. PFS significantly reduced anti-CII IgG2a and increased anti-CII IgG1 antibody level in serum from CIA mice, leading to an alteration in the ratio of IgG2a and IgG1 in favour of Th2 driven anti-CII IgG1 at 28 days after disease onset. Numorous studies have demonstrated an activated Th1 response in the absence of an adequate counterbalancing Th2 response before the clinical onset of CIA [22]. This observation further strengthens the view that PFS administration directly prevents inflammation vial switching from a Th1 to Th2 profile leads to a clinical improvement in both CIA and RA.

A large number of cytokines are active in the joints of patients with RA. It is now clear that these cytokines such as TNF-α play a fundamental role in the processes that cause inflammation and articular destruction in rheumatoid joint tissue [23] and animal experiments [24] associated with RA. It has been reported that tissue-protective cytokines such as interleukin-4 and IL-22 reduce the severity of collagen-Induced Arthritis [25–27]. IL-22 also plays a dual role that is protective prior to the onset of arthritis and pathogenic after onset of arthritis [28]. In line with these studies, the protective effects of PFS on CIA may be due to inhibiting the synthesis of proinflammatory cytokines such as TNF-α and increasing IL-4 and IL-22 in CIA after onset of arthritis. This is consistent with previous observations where we documented that PFS dampens IL-6 levels in AIA rats [21].

TLRs have important functions in innate immunity and inflammation, TLR4 signaling that involves in TNF-α and IL-6 mediated osteoclast differentiation has been validated as therapeutic targets in RA [29]. TLR4 initiates signals for different pathways that activate transcription factors like NF-κB, AP-1, leading to the production of inflammatory cytokines, and proteolytic enzymes in synovium and osteoclast formation [30,31]. TLR4 are all strongly upregulated in rheumatoid synovial tissue and RA joints. TLR4 signaling also plays a role in RA by inducing auto-antigen-specific adaptive immune responses, thereby resulting in persistent joint damage. Attenuating TLR4 signaling pathways is believed to be beneficial in RA management. In this study, we found that PFS mitigated TLR4 signaling. This was demonstrated by the suppressed protein expressions of TLR4 and p-p65 in both in CIA and RANKL-induced osteocastogenesis, and the expression of c-Fos in CIA.

The presence of activated NF-κB transcription factors has been demonstrated in human arthritic joints, the joints of animals with experimentally induced RA and Osteoclast [31,32]. Following cell stimulation by appropriate cytokines, I-κB proteins become phosphorylated, leading to its ubiquitination and proteolysis, NF-κB activation is initiated, and proinflammatory cytokine production is then induced, including TNF-α and MMPs which are key players in the pathogenesis of RA [33]. Here, the phosphorylation of p65 was depressed by PFS administration in CIA mice and RANKL-induced osteoclastogenesis, and significant inhibition activities of Periplocin on p65 phosphorylation were found in RANKL-induced osteoclastogenesis. Accordingly, we found that higher concentration of PFS significantly inhibited phosphorylation both in CIA mice and RANKL-induced osteoclast differentiation. Periplocin showed a lower activity for attenuating I-κBα phosphorylation, whether other compounds affect this signaling molecule that can impact osteoclast formation and arthritis bone resorption remains to be defined. NF-κB activation is coordinated with the degradation and synthesis of I-κB proteins [34–36]. The increased I-κBα in our CIA model might be responsible for strong negative feedback that allows for a fast turn-off of the NF-κB response, However, I-κBα levels were significantly alleviated upon PFS treatment. Previous studies have reported that STAT3 involved in numerous inflammatory diseases including RA. STAT3 could interact with NF-κB, and concomitant activation of the STAT3 and NF-κB pathways could exacerbate the inflammatory responses such as those for TNF-α and IL-6, which recruit immune cells to the inflamed synovium and pannus [37]. STAT3 also plays a pivotal role in RANKL-induced osteoclast formation, a delicately controlled balance by a bioavailable molecule can attenuate RANKL-induced osteoclastogenesis by blocking both NF-κB and STAT3 activity [38]. In accordence to these reports, an increased level of phospho-STAT3 was found in both CIA mice and RANKL-induced osteoclastogenesis treated with PFS.

Previous studies have shown that C-Fos/AP-1 is critical to Joint erosion [39] and NFATc1 is a key regulator in osteoclastogenesis [40]. NF-κB and AP-1 can bind to the NFATc1 promoter and regulate its expression. NFATc1 plays a crucial role in the regulation of osteoclastogenic marker genes during RANKL-mediated osteoclast differentiation, and it gradually induces the expression of osteoclast-specific genes, including Cathepsin K [41]. In this study, PFS treatment decreased c-Fos expression in CIA mice, and PFS abated the NFATc1 expression in RANKL-induced osteoclasts. Our data implicate that PFS could be considered as a promising agent for therapeutic intervention to alleviate inflammatory arthritis and ameliorate inflammatory focal bone erosion by interfering on the AP-1 pathway.

To explore the possible mechanism of PFS’s beneficial effects on bone and cartilage erosion, we measured two proteolytic enzyme expressions. Cathepsin K and MMPs contributes disease pathology of synovial hyperplasia and cartilage degradation in RA [42,43]. Constitutively overexpressed cathepsin K in mice leads to marked proliferative synovitis and destruction of articular cartilage and bone, and MMP-9 gene deletion mice are significantly less vulnerable to arthritis in mice experiment [44,45]. In line with the results of several reports indicating that MMP-9 are important for pannus formation. Both cathepsin K and MMP-9 is upregulated by inflammatory cytokines such as TNF-α and IL-1 [46]. Therefore, the inhibition of different cathepsin K and MMPs could also be one of the most effective therapeutic targets to prevent RA. In this study, immunohistology revealed that cathepsin K and MMP-9 expressions in paw tissue were significantly decreased in PFS treated CIA mice, and western blot analysis identified that cathepsin K expression in RANKL-induced osteoclastogenesis was inhibited by PFS treatment, and MMP-9 expression in CIA mice and RANKL-induced osteoclastogenesis was suppressed by PFS treatment. Taken together, PFS might relieve bone resorption and cartilage degradation in RA therapy.

In conclusion, oral PFS demonstrated anti-arthritic effects in vivo, and relevant activities were also evident in vitro, using the CIA mouse model. PFS effectively regulated expression of cytokines in a CIA model by reducing NF-κB signaling in vivo. PFS also suppressed osteoclastogenesis in BALB/c mice in vitro. Such effects may be due to its regulation on crosstalk between NF-κB, c-Fos/AP-1 and proinflammatory factors. Thus, we have described some of the multiple therapeutic mechanisms of PFS acting to mitigate autoimmune arthritis. This makes PFS a suitable new anti-rheumatic therapeutic candidate for further evaluation.

## Supporting information

S1 Table Histology after murine PFS treatment. (DOCX)

S2 Table Immunohistology after murine PFS treatment. (DOCX)

S1 Fig PFS downmodulate the protein expression of NF-κB pathway in the paws. (DOCX)

S2 Fig PFS and Periplocin inhibit osteoclast formation. (DOCX)
