# Supplementary material for: Periploca forrestii saponin ameliorates CIA via suppressing proinflammatory cytokines and nuclear factor kappa-B pathways
Source: PLoS One. 2017 May 2;12(5):e0176672. doi: 10.1371/journal.pone.0176672 (PMC5412996; doi:10.1371/journal.pone.0176672)
Supplement: S1 Table — (DOCX) [file pone.0176672.s001.docx]

**S1 Table. Histology after murine PFS treatment on Day 28 of collagen-induced arthritis.** Histology score represents immunization of mice with chicken type I1 collagen on day 28. Synovial infiltrates, cartilage damage, and proteoglycan depletion were scored on a scale of 0-3. Values are the mean ± SD of 20-25 experiments with at least 6 mice per group. *P < 0.05 versus vehicles, student’s t-test.

| Group | Synovial infiltrates | Cartilage damage | Proteoglycan depletion | osteoclast |
| --- | --- | --- | --- | --- |
| Control | 0.2±0.1 | 0.1±0.0 | 0.3±0.0 | 16.9±6.5 |
| Vehicle | 1.4±0.1 | 0.8±0.1 | 1.5±0.4 | 30.6±5.7 |
| PFS | 1.0±0.1* | 0.4±0.1* | 0.7±0.1* | 21.8±4.5* |
