# Supplementary material for: Periploca forrestii saponin ameliorates CIA via suppressing proinflammatory cytokines and nuclear factor kappa-B pathways
Source: PLoS One. 2017 May 2;12(5):e0176672. doi: 10.1371/journal.pone.0176672 (PMC5412996; doi:10.1371/journal.pone.0176672)
Supplement: S2 Table — (DOCX) [file pone.0176672.s002.docx]

**S2 Table. Immunohistology after murine PFS treatment on Day 28 of collagen-induced arthritis.** Immunohistology score represents immunization of mice with chicken type II collagen on day 28. STAT3, p-p65, Cathepsin K and MMP-9 were scored on ImageJ software. Values are the mean ± SD of 5 experiments with at least 6 mice per group. *P < 0.01 versus vehicles, by Student’s t-test.

| Group | STAT3 | p-p65 | Cathepsin K | MMP-9 |
| --- | --- | --- | --- | --- |
| Control | 10.8±7.0 | 41.8±23.5 | 4.2±1.5 | 27.2±18.1 |
| Vehicle | 294.4±79.7 | 416.0±32.4 | 198.0±19.8 | 395.4±108.7 |
| PFS | 154.8±56.9* | 118.6±54.0* | 75.8±31.2* | 117.4±48.2* |
