# Supplementary material for: Periploca forrestii saponin ameliorates CIA via suppressing proinflammatory cytokines and nuclear factor kappa-B pathways
Source: PLoS One. 2017 May 2;12(5):e0176672. doi: 10.1371/journal.pone.0176672 (PMC5412996; doi:10.1371/journal.pone.0176672)
Supplement: S1 Fig — (DOCX) [file pone.0176672.s003.docx]

**S1 Fig PFS downmodulate the protein expression of NF-κB pathway specifically in the paws.** On day 28, mice paws were homogenized, lysates were analyzed by western blotting with antibody against TLR4, I-κBα, p-I-κBα, p65, p-p65, STAT3, p-STAT3, c-Fos, NFATc1 and MMP-9. Protein levels were quantified using densitometry. Data represent four experiments of Mean ± SEM. *P < 0.05 **P < 0.01 versus vehicle group, student’s t-test. P: Periplocin.

*

**

**

**

*

*

**

*

**

**

**

*

**

*
