# Supplementary material for: Periploca forrestii saponin ameliorates CIA via suppressing proinflammatory cytokines and nuclear factor kappa-B pathways
Source: PLoS One. 2017 May 2;12(5):e0176672. doi: 10.1371/journal.pone.0176672 (PMC5412996; doi:10.1371/journal.pone.0176672)
Supplement: S2 Fig — (DOCX) [file pone.0176672.s004.docx]

**S2 Fig PFS and Periplocin inhibit osteoclast formation in BALB/c mice**. **(A)** Chemical structure of Periplocin. **(B)** Osteoclast were cultured as method in Osteoclastogenesis in the presence or absence of PFS and Periplocin in the indicated concentrations. Cell lysates were analyzed by western blotting with antibody against TRL4, I-κBα, p-I-κBα, p65, p-p65, STAT3, p-STAT3, c-Fos, NFATc1, MMP-9 and Cathepsin K**.** Protein levels were quantified using densitometry**.** Data represent triple experiments. Mean ± SEM. *p<0.05, **p<0.01 versus M-CSF + RANKL, student’s t-test. P: Periplocin.

 **(A)**

**(B)**

**

**

**

**

*

*

*

**

**

**

*

*

*

*

*
